# The assessment value of transcranial Doppler sonography versus magnetic resonance angiography in vertebrobasilar stroke

**Published:** 2010

**Authors:** Abbas Ghorbani, Fereshteh Ashtari, Farzad Fatehi

**Affiliations:** a Neurology Department, Isfahan University of Medical Sciences, Isfahan, Iran; b Isfahan Neuroscience Research Center, Isfahan, Iran; c Isfahan Medical Education Research Center, Isfahan, Iran

**Keywords:** Transcranial Doppler, Brain MRI, Brain MRA, Vertebrobasilar Stroke, Stenosis

## Abstract

**BACKGROUND:**

The goal of this study was to determine the reliability of TCD in evaluation of vertebrobasilar arteries in comparison with brain MRA in patients suffering from acute vertebrobasilar stroke.

**METHODS:**

Samples were patients with definite clinical diagnosis of vertebrobasilar stroke. For all patients brain MRI, MRA and TCD were performed during the first 48 hours of admission. Basilar artery was insonated at the depth of 75 to 85 mm and vertebral arteries were insonated at the depth of 45 to 55 mm. On brain MRA, the degree of stenosis in vertebrobasilar arteries was graded from I (normal) to IV (total stenosis) and the correlation between the grade of stenosis and TCD indices were studied.

**RESULTS:**

Spearman correlation test revealed a significant correlation between mean flow velocity (MFV) and MRA grading (correlation coefficient = -0.486) as well as end diastolic velocity (EDV) and MRA grading (correlation coefficient = -0.323) with no significant correlation between pulsatility index, peak systolic velocity and MRA grading (p > 0.05). One way ANOVA analysis showed that there was only significant mean MFV and mean EDV difference between grade 1 and other grades.

**CONCLUSIONS:**

TCD was only able to differentiate between stenotic and normal pattern and could not assist in the grading of stenosis. On the other hand, in acute vertebrobasilar stroke that TCD performed blindly without visualization of arteries and in a fixed depth it might have limited value in the grading of vertebrobasilar system stenosis.

Transcranial Doppler (TCD) since its introduction in 1982, has become widely utilized to examine the basal cerebral arteries for different diseases as a result of its portability, low price, and swift noninvasive nature; in other words, it has been rapidly developing from a simple noninvasive diagnostic tool to an imaging modality with an extensive continuum of clinical applications.^1^–^5^

Vertebrobasilar stroke presents with characteristic manifestations attributable to impaired perfusion of the cerebellum, the brain stem, and the occipital cortex awing to reduced perfusion generally caused by atherosclerosis or thromboembolism and the choice of treatment depends on understanding the diverse causal pathophysiologic mechanisms.^6^ After diagnosing vertebrobasilar ischemia by taking history and physical examination, the evaluation of vertebrobasilar arteries is of paramount importance since understanding the offending pathology of vertebrobasilar ischemia (i.e. small vessels disease versus embolic or large arterial thrombosis) may assist in decision making for further treatment. One noninvasive way to assess the posterior circulation system is utilizing magnetic resonance angiography (MRA); however, it is costly and also in acute setting time consuming. Noticeably, TCD is a non-invasive method which could be applied on bed side.^7^ The goal of this study was to determine the reliability of TCD in evaluation of vertebro-basilar arteries in comparison with MRA in the patients suffering from acute vertebrobasilar stroke.

## Methods

This analytical study was performed from January 2008 to August 2009 in Al-Zahra Hospital of Isfahan University of Medical Sciences. Eligible patients with definite clinical diagnosis of acute vertebrobasilar ischemia were included: patients with at least two symptoms of dysphagia, dysarthria, diplopia, dysmetria, vertigo, ataxia, crossed facial and extremities weakness or sensory deficit. In addition, patients with one of the above symptoms but a cardiovascular risk factor like hypertension, hyperlipidemia, diabetes mellitus, and ischemic heart disease were included too. For all patients brain MRI and MRA were performed during the first 48 hours of admission and patients with normal brain MRI or patients with suspected dissection of vertebro-basilar system, vasculitides, sickle cell anemia, post-angiographic stroke, infectious causes and hemorrhagic infarcts or any other non-vascular lesions explaining the symptoms were excluded. Total 27 eligible patients were included which comprised 81 arteries (27×2 vertebral arteries plus 27 basilar arteries); in other words, the total pooled arterial sample size for comparison between TCD indices and brain MRA grading was 81. For all patients, TCD was performed in the first 48 hours with transforaminal window using a 2 MHz Doppler probe (multi Doppler probes, Sipplingen, Germany) through the foramen magnum from the top of the neck below the occipital protuberance. Basilar artery was insonated at the depth of 75 to 85 mm and the extracranial segments of the vertebral arteries were insonated at the depth of 45 to 55 mm at the level of C1 vertebra. TCD was performed by neurologists blinded to the brain MRA results (most of TCDs were performed before taking MRI) and parameters such as mean flow velocity (MFV), pulsatiliy index (PI), peak systolic velocity (PSV), and end diastolic velocity (EDV) were recorded.

On brain MRA, the degree of stenosis in vertebral and basilar arteries was determined by further grading: grade IV, no visible artery; III, severe stenosis (≥ 50% stenosis in comparison with normal side); II, mild stenosis (< 50% stenosis in comparison with normal side); and I, normal arterial diameter.

The correlation between the grade of stenosis and TCD indices including MFV, PI, PSV, and EDV were calculated by spearman correlation test. Moreover, the sensitivity and specificity of TCD was studied by means of a receiver operating characteristic (ROC) curve. Considering patients’ MRA grading of two vertebral arteries and basilar artery as the reference standard, area under the ROC curves was calculated for output variables and the point of best performance was recognized as the indicator of cutoff values for all arteries and for each artery (i.e. right and left vertebral artery and basilar artery) separately. In all cases, a level of p < 0.05 was considered to be statistically significant.

## Results

**Figure 1:**
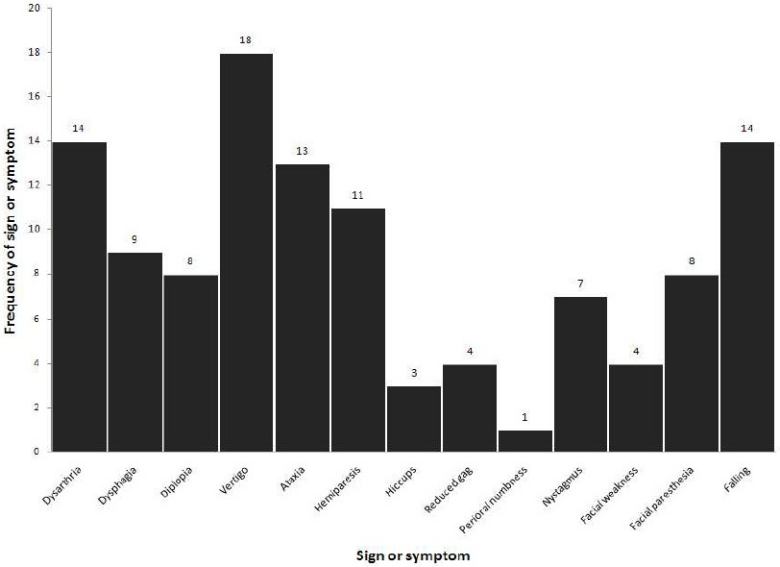
Presenting manifestations of the patients

The mean (SD) age of patients was 65.48 (10.29) including 10 (37.04%) females and 17 (62.96%) males. Nine (33.33%) patients suffered from diabetes mellitus, 16 (59.26%) from hypertension, 10 (37.04%) from hyperlipidemia, 5 (18.52%) from smoking and 5 (18.52%) from IHD. The major neurological presenting symptoms were vertigo, limb weakness and dysarthria; presenting manifestations are demonstrated in figure 1.

According to the location of infarct, medullary infarct was seen in 14 (51.9%) patients, pontine infarct was detected in 15 (55.6%), midbrain infract in 5 (18.52%), cerebellar infarct in 6 (22.22%), and occipital infarct in 5 patients (18.25%).

Spearman correlation test revealed that there was a significant correlation between MFV and MRA grading (correlation coefficient = -0.486, p = 0.000) as well as EDV (correlation coefficient = -0.323, p = 0.006) and MRA grading; however, no significant correlation between PI, PSV and MRA grading was detected (p > 0.05).

**Figure 2:**
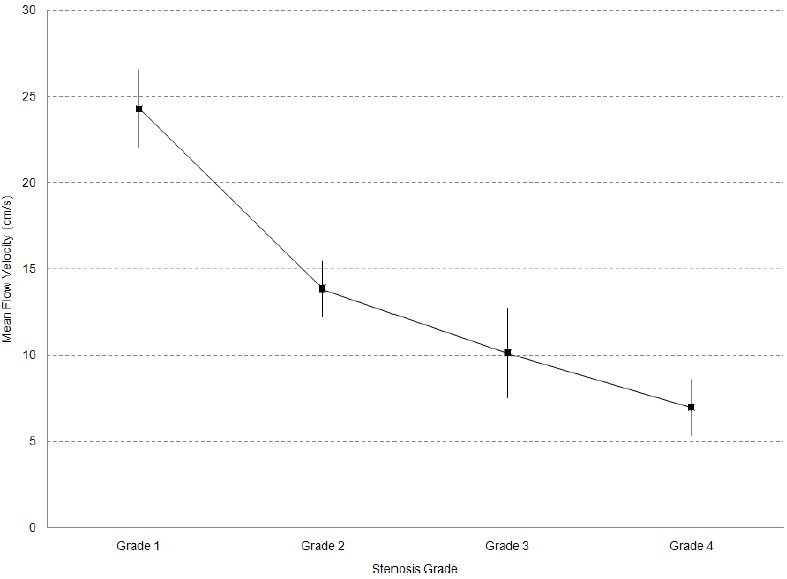
Mean MFV based on vascular grading

**Figure 3:**
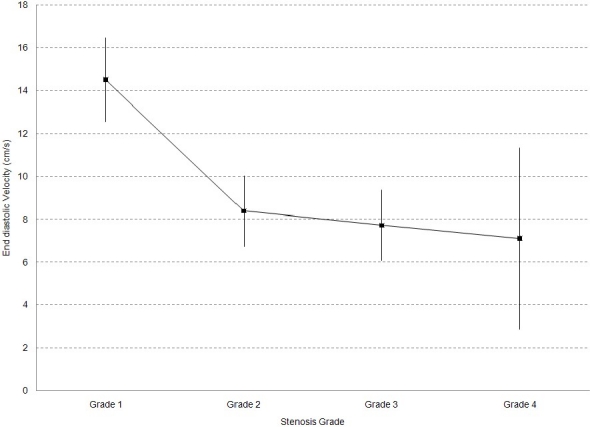
Mean EDV based on vascular grading

One way ANOVA analysis showed that there was a significant mean difference of MFV between different grades of stenosis (p value = 0.000); however, post hoc analysis demonstrated that there was not significant mean MFV difference between grades 2, 3 and 4 and only there was a significant difference between grade 1 and other grades (p value < 0.05) (Figure 2). In addition, regarding EDV, one way ANOVA analysis demonstrated that there was a significant mean EDV difference between different grades of stenosis (p value = 0.04); however, post hoc analysis demonstrated that like MFV, there was just a significant difference between grade 1 and other grades (p value < 0.05) (Figure 3).

According to the best performance point on the ROC curve, cutoff value for MFV was calculated as 15.5 cm/s for differentiating grade 1 (normal artery) from other grades (sensitivity = 0.72, specificity = 0.82); in addition, the area under ROC curve was 0.803.

**Figure 4:**
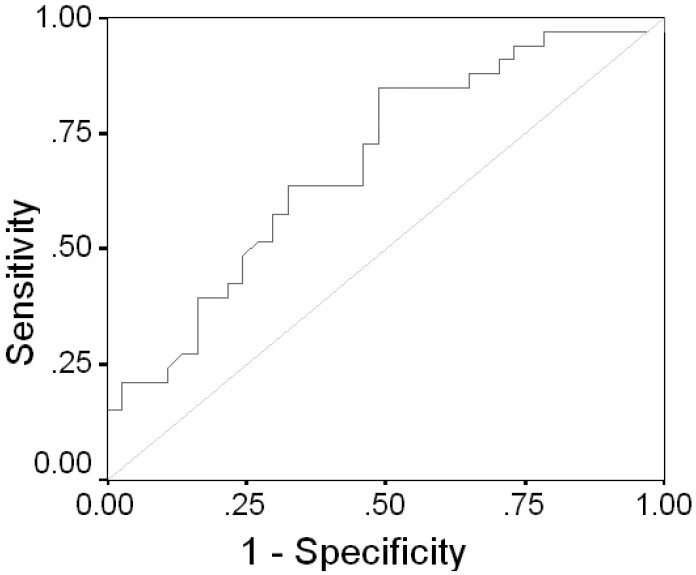
ROC curve of EDV (normal vessel diameter versus other grades)

Likewise, cutoff value for EDV was calculated as 5.01 cm/s for differentiating grade 1 (normal artery) from other grades (sensitivity = 0.85, specificity = 0.51) (Figure 4); in addition, the area under ROC curve was 0.687 (Figure 4).

Separately estimations for above cutoff values for each artery are mentioned in table 1.

**Table 1:** Separate estimation of cutoff values along with sensitivity and specificity in vertebrobasilar arteries

	Right vertebral a.	Left vertebral a.	Basilar a.
MFV cutoff point (cm/s)	12.50	20.50	18.00
Sensitivity	0.63	0.60	0.74
Specificity	0.30	0.73	0.88
EDV cutoff point (cm/s)	4.31	11.40	9.75
Sensitivity	0.88	0.60	0.80
Specificity	0.40	0.73	0.89

## Discussion

Many studies have been performed to determine the sensitivity and specificity of TCD in different situations like arterial stroke, cerebral venous thrombosis, subarachnoid hemorrhage, intensive care unit and sickle cell disease with various results.^8^–^18^ In one of the earliest studies, Sloan et al^19^ demonstrated that TCD is a highly specific (100%), but less sensitive (58.6%) test for the recognition of angiographic vasospasm following subarachnoid hemorrhage.

In another survey, Razumovsky et al^12^ compared the accurateness of TCD, MRA, and MRI in patients with acute cerebral ischemia; TCD showed a sensitivity of 96% and a specificity of 33% for recognizing abnormal cerebral blood flow velocities. MRA showed a sensitivity of 46% and a specificity of 75% for assessing intracranial vascular anatomy, whereas initial MRI revealed a sensitivity of 84% and a specificity of 100% for evaluation of ischemic parenchymal changes.

De Bray et al^8^ assessed the value of TCD versus angiography in vertebrobasilar intracranial stenoses. The sensitivity of TCD using a peak systolic frequency shift in diagnosing stenoses reached 80% and its specificity was 97% providing that only atheromatous stenoses were considered.

Navarro et al^11^ carried out a systematic review to evaluate the precision of TCD in comparison with angiography for the diagnosis of 50% or higher middle cerebral artery (MCA) stenosis in patients with transient ischemic attack or ischemic stroke. They included studies published on TCD accuracy from 1982 to the end of December 2005 using angiography as the gold standard. For 80 cm/s cutoff value of MCA flow, mean weighted average sensitivity of 92%, specificity of 92% were obtained. For 100 cm/s cutoff, the sensitivities were 100% and specificity was 97%.

In a recent large study for the evaluation of proximal vertebral artery stenosis by color Doppler imaging, PSV of origin was the most helpful hemodynamic parameter, having accuracy of 94.5%, 96.2%, and 88.7% for the diagnosis of < 50%, 50-69%, and 70-99% stenosis.^20^

The present study did have some major limitations. It is important to note that many variations in posterior circulation exist and as a result, the findings need to be argued cautiously to avoid under or over diagnosis. Another important point to be emphasized is that in vertebrobasilar stenosis, most stenoses are located in V1 segment of vertebral artery or in origin, and additionally, with the application of TCD in a fixed depth, as it was done here, one is blind to the location of stenosis; for instance, the measured TCD parameters belonged to pre-stenotic, stenotic or post stenotic portion of the artery and accordingly, the correlation estimation between TCD parameters and MRA findings exceedingly suffered from lack of concordance between the real site of lesion (in MRA) and measured parameters in TCD; therefore, it seems plausible that one starts insonation from the origin of artery and increases the depth to investigate the entire length of artery especially if performed with the aid of newer techniques such as transcranial color coded duplex ultrasonography. It goes without saying that in routine Doppler sonography with the augmentation of stenosis, parameters such as PSV, MFV, PI, and EDV rise as well but in this study since it was far from stenotic site and mostly post stenotic portions were insonated, therefore, such results were not attained. In other words, considering the present MFV results, it could be seen that with the increment of stenosis, the MFV has declined proportionally and retrospectively, it could be concluded that most measurements have not been in stenotic portion of artery (as was confirmed on brain MRA); and finally, TCD could not assist in the grading of stenosis.

## Conclusions

In conclusion, in acute vertebrobasilar stroke, according to the present findings as well as the method performed, if TCD carried out blindly without visualization of arteries and in a fixed depth, it might have limited value in the grading of vertebrobasilar system stenosis.
